# Performance of an adapted household food insecurity access scale in measuring seasonality in household food insecurity in rural Ethiopia: a cohort analysis

**DOI:** 10.1186/s40795-019-0323-6

**Published:** 2019-11-20

**Authors:** Bereket Yohannes Kabalo, Seifu Hagos Gebreyesus, Eskindir Loha, Bernt Lindtjørn

**Affiliations:** 1 School of Public Health, Wolaita Sodo University, PO Box 126, Wolaita Sodo, Ethiopia; 2 School of Public and Environmental Health, Hawassa University, Hawassa, Ethiopia; 3 Centre for International Health, University of Bergen, Bergen, Norway; 4 School of Public Health, Addis Ababa University, Addis Ababa, Ethiopia; 5 Department of Infectious Disease Epidemiology, London School of Hygiene and Tropical Medicine, London, England, UK

**Keywords:** Food insecurity, Internal consistency, Parallelism, Dose-response relationship

## Abstract

**Background:**

Seasonality poses a considerable food security challenge in Ethiopia. Yet, measuring seasonal variations in food insecurity, particularly the dimension of food access, lacks an adequately validated tool. We therefore evaluated the performance of an adapted Household Food Insecurity Access Scale (HFIAS) to estimate seasonal variations in food insecurity (FI) among subsistence villagers in Ethiopia.

**Methods:**

We employed a cohort study design using a panel of four repeated measurements taken in June, September, and December in the year 2017, and in March 2018. The study recruited 473 villagers from the drought-affected Wolaita area in southwest Ethiopia. The performance of the HFIAS was evaluated via internal consistency (Chronbach’s alpha values) and criterion validation techniques. The set of criteria include: parallelism between affirmative responses to FI questions and wealth strata; dose-response relationship between FI and dietary intake; and also FI severity and household wealth status.

**Results:**

This study revealed that the HFIAS had satisfactory performance in four repeated measurements. The likelihood of affirmative responses to questions about FI decreased with ascending wealth quintiles. We observed an inverse dose-response relationship between FI and wealth status, and between FI and household dietary diversity.

**Conclusions:**

The HFIAS showed an acceptable potential for measuring seasonal variations in FI in the study area. Our findings complement efforts to evaluate the scale’s applicability in various settings, in order to promote cross-culture monitoring and comparisons. However, it required a careful adaption for contextual and cultural sensitivities.

## Background

The Food and Agricultural Organization (FAO) of the United Nations defines household food security as access to a diet of sufficient quantity and quality for all household members at all times, through socially acceptable ways [1]. Yet, more than 800 million people globally still suffer from lack of sufficient food, food insecurity (FI) [2]. About 30% of this burden occurs in Sub-Saharan Africa and Ethiopia contributes the major share [2, 3].

Food security measurement has four common dimensions: availability, access, utilization, and stability of the other three dimensions over time [1]. Although widely used, food availability often lacks accuracy in quantifying how adequately food is distributed at household and individual levels [2]. Even when food stocks are sufficient, food access may still remain a challenge [4, 5]. Food utilization dimension applies non-specific indicators that do not take into account subjective experiences of households [6–8].

Given the multidimensional nature and its complex components, FI measurement has still remained subject to debate [2]. Accordingly various indicators of FI measurement have been developed over decades [9]. The widely used indicators often involve either dietary diversity and food frequency, or consumption behaviours at different levels [4, 10–14]. Moreover, studies have shown that FI results from the different indicators also tend to be comparable [10, 15], yet the overall estimates still show variations [15, 16]. Nonetheless, developing a single comprehensive measure for FI dimensions has continued to be an going challenge [16].

The Household Food Insecurity Access Scale (HFIAS) has been used to measure food access dimension of FI [17]. This tool comprises nine questions in three domains that describe core experiences: uncertainty or anxiety about food supply; insufficient food quality; and insufficient food intake and its physical consequences [18]. The HFIAS tool is intended to capture a mix of food insufficiency and psychological factors related to FI, but some researchers raise its subjectivities such as response biases [19]. Yet, few studies have evaluated the validity of the HFIAS in developing countries such as Ethiopia. Despite limited evidence on its validity, this tool has been frequently used to measure FI in many settings [20–23]. Recently, Gebreyesus et al. reported that the tool had satisfactory validity in the Butajira district in central Ethiopia [23]. However, their cross-sectional analysis did not assess the HFIAS tool’s performance across agricultural seasons. To be effective, validation of tools like the HFIAS should account for seasonal variations in food insecurity [18, 20].

The densely populated Wolaita area in southwest Ethiopia has been repeatedly affected by drought [24, 25]. However, little is known about the risk profile of this area across agricultural seasons, and thus a valid measuring tool is needed. We therefore did a panel study aimed to assess how well the HFIAS measures seasonal variations in FI among subsistence farming households. The data analyzed for this study were extracted from a larger longitudinal study assessing seasonal variations in food and nutrition insecurity in the area.

The findings of this study are expected to improve our knowledge of the applicability of the HFIAS for use in developing countries, such as Ethiopia. We also expect that the findings might contribute as input to the body of literature on FI measurements.

## Methods

### Study design

We employed a cohort study design by collecting data from a panel of 473 rural households in the Wolaita area. Rural villages in the area represent two agro-ecological divisions: Lowlands (with hot and semi-dry conditions) and Midlands (with relatively cooler and sub-humid conditions) [5, 26]. The study involved same participants at four data points in time (rounds): June 2017, September 2017, December 2017, and March 2018.

### Study setting

This study was conducted in two rural districts (Woredas), namely Humbo and Sodo Zuria in the Wolaita area [25, 27]. About 400,000 people live in the districts often suffering from chronic food insecurity problem [5, 28]. The households were recruited from lowland (< 1600 m) and midland (> 1600 m) areas.

Based on the amount and timing of seasonal rains, farming activities, crop harvest, and other area-specific contexts, we identified and accounted for four distinct agricultural seasons in our survey rounds [25, 29]. Survey round 1 (R_1_) was conducted in the month of June in the heavy rainy season. The second round (R2) was conducted in September when the main cereal crop harvest takes places; small rains in this season give opportunity for growing root varieties. The third round (R3) was conducted in December, a late post-harvest month. The dry season lasts from late December to February or March. The fourth round (R4) was conducted late in the dry season; the second rainy season. Accordingly R1 and R4 were in pre-harvest season, R2 was at main harvest and R3 late in post-harvest seasons [5, 25, 30].

### Study participants

As a subsample of the broader study, the current analysis included 473 households who were not taking part in the PSNP. We excluded households taking part in the programme [31, 32]. As the programme member households get periodic cash support, their income basis would differ from households not taking part in the programme [31, 33, 34]. We further intended to maintain comparability with previous studies [21–23].

### Study instrument

We adapted the HFIAS questionnaire, which was recently validated in the rural Butajira district in central Ethiopia [23]. The tool comprises nine questions which are based on the respondent’s recall of food insufficiency and related psychological responses in the past 30 days [4, 18]. The questions (and their shortened versions) are as follows:

- Q_1_: ‘Did you worry that your household would not have enough food?’ (‘Worry for food’)
- Q_2_: ‘Were you or any household member not able to eat the kinds of foods you preferred because of a lack of resources?’ (‘Unable to eat preferred foods’)
- Q_3_: ‘Did you or any household member eat just a few kinds of food day after day because of a lack of resources?’ (‘Eat a limited variety of foods’)
- Q_4_: ‘Did you or any household member eat food that you did not want to eat because of a lack of resources to obtain other types of food?’ (‘Eat foods that you did not want’)
- Q_5_: ‘Did you or any household member eat a smaller meal than you felt you needed because there was not enough food?’ (‘Eat a smaller meal’)
- Q_6_: ‘Did you or any household member eat fewer meals in a day because there was not enough food?’ (‘Eat fewer meals in a day’)
- Q_7_: ‘Was there ever no food at all in your household because there were no resources to get more?’ (‘No food to eat of any kind’)
- Q_8_: ‘Did you or any household member go to sleep at night hungry because there was not enough food?’ (‘Go to sleep at night hungry’)
- Q_9_: ‘Did you or any household member go a whole day without eating anything because there was not enough food?’ (‘Go day and night without eating’); the capital letter ‘Q’ denotes a question and subscripts 1–9 are item numbers in increasing severity. Each question in the tool includes a follow-up item to determine the frequency of occurrence whose responses are coded as often ‘3’, sometimes ‘2’, rarely ‘1’, or not at all ‘0’

A language expert and one of the investigators (BYK) translated the questionnaire into the local Wolaita language. We then interviewed five women in lowland and five in midland areas to ensure that each item was understandable and not easily misinterpreted. These women were later not included here in the main analysis. We asked each woman all the nine questions and recorded their responses. Afterwards, each woman was asked if she understood a particular question. When limitations that could compromise the intended meanings were noted, we asked the women how such items could be improved. Finally, the 10 respondents, two field supervisors, and one of the investigators (BYK) discussed the results, and finally BYK compiled these into a modified module [23].

The preliminary test of the HFIAS revealed several contextual and cultural sensitivities. For example, all the 10 women answered ‘yes’ to Q_1_ (‘worry for food’). They identified the term ‘worry’ in Q_1_ as an ordinary situation, this required contextual modification. Respondents also described Q_4_ (‘Eat foods that you did not want’) as intrusive and embarrassing, thus requiring the addition of a brief discussion about area-specific food taboos, including a list of foods that are consumed only during extreme food shortages. Some of the women were shy or afraid of replying ‘yes’ for Q_7_ (‘No food to eat of any kind’), Q_8_ (‘Go to sleep at night hungry’), and Q_9_ (‘Go day and night without eating’). This could be due to religious perceptions that disclosing such extreme situations would be perceived as insubordination to God. These three items thus required a focused training to interviewers on probing skills. For this to be effective, having interviewers who were better aware of extreme food shortages conditions had a particular importance.

### Data collection

Ten data collectors and two supervisors who are native speakers of the local language were recruited and given training on the different modules of the questionnaire. The same data collectors (in most cases) interviewed the same respondents in all the four rounds. However, in some rare cases a similarly trained one data collector was substituted. Women were mainly recruited as respondents in this study. However, when a woman was unavailable, any adult who was present and ate food in the household in the previous day was asked. Women are commonly responsible for food preparation and child feeding roles in their households in the study area [23, 35, 36]. Moreover, the women commonly remain at home more often than any other family member in this area.

### Outcome measure

The household food insecurity was measured by using responses to the nine FI occurrence questions (Q_1_-Q_9_) and their follow-up frequency of occurrence items [18, 23]. Accordingly, households were grouped into four categories (levels). A household is food-secure if it scored ‘0’ or ‘1’ in the first FI frequency of occurrence question and ‘0’ in Q_2_ to Q_9_; Mildly food-insecure if the first FI frequency of occurrence item has ‘2’ or ‘3’ or the second item has ‘1,’ ‘2,’ or ‘3’ or the third item has ‘1’ or the fourth item has ‘1’ and items Q_5_ to Q_9_ score ‘0’; Moderately food insecure if item three = ‘2’ or ‘3’ or item four = ‘2’ or ‘3’ or item five = ‘1’ or ‘2’ or item six = ‘1’ or ‘2’ and item seven to nine = ‘0’; and.

Severely food insecure if item five = ‘3’ or item six = ‘3’ or item seven = ‘1,’ ‘2,’ or ‘3’ or item eight = ‘1,’ ‘2,’ or ‘3’ or item nine = ‘1,’ ‘2,’ or ‘3’ [18, 37].

The overall household FI prevalence was computed as the proportion of food-insecure households out of the total interviewed. The mean differences of each consecutive pair of data time points were considered to estimate seasonal variations of the outcome measure.

### Wealth measure

A wealth index was constructed using a principal component analysis of the data on household-level assets. These assets included the housing structure (upper most cover, interior roof, floor, and wall) based on construction materials as observed by the interviewers, as well as possession of items such as radios, mobile telephones, beds, mattresses, kerosene lamps, watches, electric or solar panels, chairs, tables, wooden boxes, and carts. The four components of the housing structure had ordinal responses ranked mostly from 0 to 3. However, household assets had responses from 0 to 1 only. We standardized these scores to reduce the tendencies that variables with greater response would underestimate the others. Through a dimension-reduction analysis these inventories with standardized scores were summarized into logical dimensions. Based on standardized scores, the households were lastly categorized into relative wealth quintiles: Poorest (20th percentile), Poor (40th percentile), Medium (60th percentile), Rich (80th percentile), and Richest (>80th percentile).

### Dietary intake

The dietary diversity was measured by using Household Dietary Diversity Scale (HDDS) [38]. The HDDS was derived from previous day’s consumptions of households based on 12 food groups; ranging between 0 and 12 scores [39, 40]. Respondents were asked qualitatively about their entire households’ food intake in the 24 h preceding each data collection round of the survey, focusing on consumption of 12 food groups: (i) meat; (ii) fish; (iii) vegetables; (iv) fruits; (v) eggs; (vi) potatoes and other roots or tubers; (vii) dairy products; (viii) pulses (ix) cereals and breads; (x) oil, fat, or butter; (xi) sugar or honey; and (xii) other foods, such as coffee and tea [23]. When the respondent was asked if her family had diet from a particular food group, cereal for example, she would reply either ‘yes’ or ‘no’. Accordingly a ‘yes’ response was coded as ‘1’ and if ‘no’ it was coded as 0. The sum of the ‘yes’ response codes for the 12 food groups gives us the HDDS.

### Statistical analysis

We used SPSS software (version 25 Inc., Chicago.IL) and Stata (Version 14, Stata Corporation, College Station, TX) for data analyses. Reliability analysis was conducted to estimate Chronbach’s alpha values. Likelihoods of affirmative responses were evaluated for parallelism across wealth quintiles. Extended Mantel-Haenszel chi square for linear trend was used to check for dose-response relationships between wealth and FI strata. Reproducibility of item responses was evaluated for pairs of related seasons (between pre-harvest seasons and also between post-harvest seasons) through paired *t*-test for equality of means of the HFIAS scores. We applied one-way analysis of variance (ANOVA) with robust tests of equality of means for multiple comparisons [41, 42].

### Factor analysis

We did an exploratory analysis involving the nine items through a varimax rotation of responses. A Horn’s parallel analysis (PA) was used to determine the number of factors to retain based on observed eigenvalues compared with that obtained from uncorrelated normal variables.

### Validation

The following criteria were used for validation of the nine HFIAS tool: Chronbach’s alpha values approaching 0.85 to assert internal consistency [21–23]; parallelism of item-responses across wealth quintiles; and the presence dose-response relationship between wealth and FI strata, and between dietary intake and food security [41, 42].

## Results

### Baseline characteristics

We included 473 households in the analysis. The mean age of respondents was 30.5 years (standard deviation of 9.2), and 95.6% (452) were married women. The majority 81.4% (385) were able to read or write. About 70.0% (331) of households worked exclusively as farmers and the rest earned additional income as labourers in nearby towns. Among households, 25.8% (122) participated in local microfinance packages, 13.7% (65) had no farming plots and 58.8% (278) had less than a hectare. Households also owned assets such as radio (28.1%); mobile phone (57.9%); beds and mattress (45.7%); chairs or tables (70.8%). Table 1 further describes household size and livestock ownership.

**Table 1:** Baseline socio-economic characteristics of sample households in rural Wolaita, Ethiopia

Characteristics (*N* = 473)	Category	# (%)
Household size	8^+^	120 (25.4)
	5–7	217 (45.9)
	<=4	136 (28.7)
Livestock (#)		
Cattle	0	91 (19.2)
	1	126 (26.6)
	2	127 (26.8)
	3	55 (11.6)
	> = 4	74 (15.6)
Calves	0	151 (31.9)
	1	195 (41.2)
	> = 2	127 (26.8)
Chickens	0	175 (37.0)
	1–2	132 (28.0)
	3–4	120 (25.3)
	> = 5	46 (9.7)
Goats	0	310 (65.5)
	> = 1	163 (34.5)
Sheep	0	354 (74.8)
	> = 1	119 (25.2)
Donkeys	0	367 (77.6)
	> = 1	106 (22.4)

### Responses to household food insecurity access scale questions

The overall affirmative responses (‘yes’) to the nine HFIAS items were found to be sequentially ordered in increasing severity, with some internal deviations. Similarly, the pre-harvest rounds (R1 and R4) had relatively higher affirmative response ranges for the nine questions (Q_1_–Q_9_). If we take the proportion of affirmative response for Q_1_ it was 9.1% in June (R1) and 17.8% in March (R4) whereas 5.9% in September (R2) and 8.0% in December (R3). Similarly it was 75.3% in R1 and 86.5% in March (R4), compared to 64.9% in R2 and 60.5% in R3. Accordingly, Q_1_ (‘Worry for food’) received the highest affirmative responses, except in R3. The ninth item Q_9_ (‘go day and night without eating anything’) had the fewest affirmative responses across all four rounds. In the third round, Q_4_ (‘Eat foods that you did not want’) had the highest affirmative responses, and Q_6_ (‘eat fewer meals in a day’) had more affirmative responses than Q_5_ (‘Eat a smaller meal’). Similarly, in Round 4, the fourth item (Q_4_) had a slightly higher response than its preceding item: Q_3_ (‘eat a limited variety of foods’). Table 2 summarizes the results.

**Table 2:** Affirmative responses to items on the Household Food Insecurity Access in rural Wolaita area, Ethiopia (*N* = 473)

Household Food Insecurity Access Scale (HFIAS) questions	Data collection times			
	June 2017 (Round 1) ^pre^	Sept. 2017 (Round 2) ^post^	Dec. 2017 (Round 3) ^post^	March 2018 (Round 4) ^pre^
	# (%)	# (%)	# (%)	# (%)
Q_1_. Worry for food	356 (75.3)	307 (64.9)	286 (60.5)	409 (86.5)
Q_2_. Unable to eat preferred foods	314 (66.4)	280 (59.2)	282 (59.6)	367 (77.6)
Q_3_. Eat a limited variety of foods	303 (64.1)	252 (53.3)	262 (55.4)	358 (75.7)^a^
Q_4_. Eat foods that you did not want	292 (61.7)	233 (49.3)	298 (63.0)^a^	359 (75.9)^a^
Q_5_. Eat a smaller meal	279 (59.0)	194 (41.0)	179 (37.8)^a^	352 (74.4)
Q_6_. Eat fewer meals in a day	230 (48.6)	184 (38.9)	215 (45.5)^a^	292 (61.7)
Q_7_. No food to eat of any kind	97 (20.5)	77 (16.3)	119 (25.2)	162 (34.2)
Q_8_. Go to sleep at night hungry	48 (10.1)	41 (8.7)	60 (12.7)	101 (21.4)
Q_9_. Go day and night without eating	43 (9.1)	28 (5.9)	38 (8.0)	84 (17.8)
Overall prevalence of household food insecurity (95% CI)	71.0 (66.9–75.1)	61.1 s (56.7–65.5)	78.9 (75.2–82.6)	86.3 (83.1–-89.4)

Q_1_ to Q_9_ are serial numbers of the items in the scale in order of severity

*CI* confidence interval, ^*Pre*^ Pre-harvest, ^*post*^ Post-harvest

^a^Items with deviations in affirmative responses

Affirmative responses to the Q_1_–Q_6_ were higher in R1, decreased in R2, increased again in R3, and peaked in R4. However, responses to Q_7_–Q_9_ had slight differences across the first three rounds. Accordingly, March and June showed the highest affirmative responses and September showed the lowest.

**Fig. 1:**
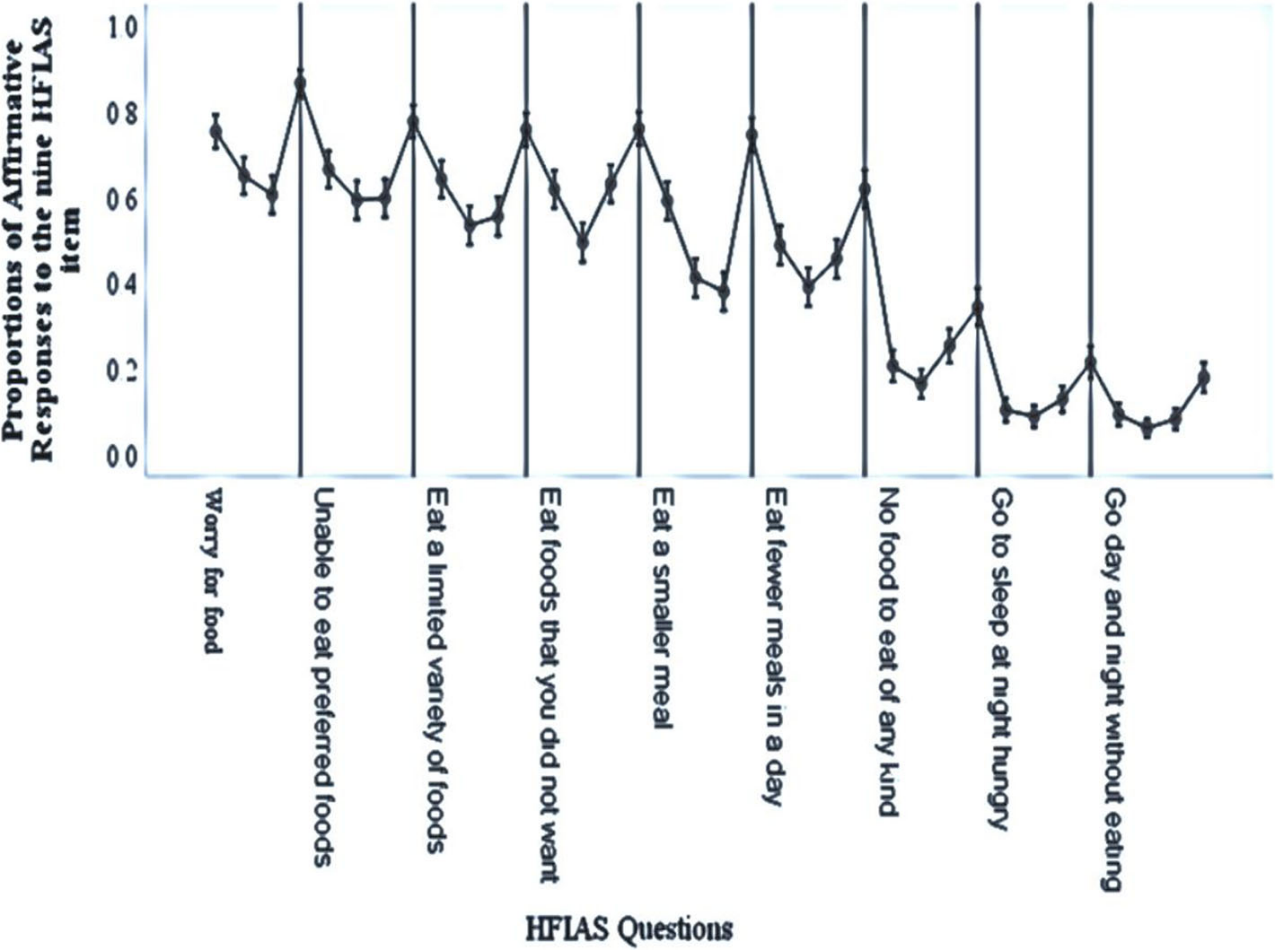
Variations in affirmative responses to the nine HFIAS questions across rounds, rural Wolaita, Ethiopia

Figure 1 shows the highest and lowest oscillations of affirmative responses to each of the nine items in separate boxes, parallel with the Y-axis. The four error bars for each item representing the rounds are in the following order: June (R1), September (R2), December (R3), and March (R4). The vertical line after each item (item box) separates the item from its preceding or subsequent one. For example, the vertical line next to the main Y-axis separates the item ‘worry for food (Q_1_)’ and the subsequent item ‘unable to eat preferred foods (Q_2_)’; an error bar on the line itself also belongs to the first item (% of affirmative response to Q1 during R4). Accordingly, the four error bars (3 before the line and 1 overlapping on the line) indicate the item just before the vertical line. The area before the vertical line together with the line itself is meant to show the particular item as a single entity; how affirmative responses to the item oscillate across the four rounds (R1 through R4). As shown in the figure, item responses fluctuated across the four data collection rounds, particularly during pre-harvest (R1 and R4) and post-harvest rounds (R2 and R3).

### Construct validity

Based on the exploratory factor analysis, two dimensions were consistently retained in the model. The first factor (F1) loaded most on the first six questions and the second factor (F2) on the last three questions. Accordingly, F1 implies mild-to-moderate FI and F2 implies severe FI (Table 3). The total combined variance explained by the two factors in response was 83.4% in R1, 82.9% in R2, 71.6% in R3, and 81.7% in R4. The total combined variance in response from pooled observations of the four rounds was 75.5%.

**Table 3:** Factor loading for rotated component matrix of responses to the nine Household Food Insecurity Access Scale items, rural Wolaita, Ethiopia (*N* = 473)

HFIAS questions	Survey rounds									
	Round 1		Round 2		Round 3		Round 4		Pooled	
	F1	F2	F1	F2	F1	F2	F1	F2	F1	F2
Q1. Worry for food	.82	.05	.90	.00	.55	.07	.78	.07	.76	.04
Q2. Unable to eat preferred foods	.95	.11	.95	.08	.68	.03	.94	.14	.88	.09
Q3. Eat a limited variety of foods	.96	.14	.93	.21	.23	.70	.93	.16	.83	.19
Q4. Eat foods that you did not want	.95	.18	.89	.32	.84	.28	.94	.16	.92	.18
Q5. Eat a smaller meal	.93	.23	.75	.49	.88	−.25	.84	.22	.86	.16
Q6. Eat fewer meals in a day	.76	.42	.71	.54	.75	.27	.76	.40	.75	.38
Q7. No food to eat of any kind	.27	.86	.26	.85	.16	.77	.30	.85	.26	.87
Q8. Go to sleep at night hungry	.10	.89	.12	.80	.03	.74	.13	.93	.12	.90
Q9. Go day and night without eating	.09	.87	.08	.71	−.07	.68	.10	.90	.09	.84

Extraction method: principal component analysis. Rotation method: Varimax with Kaiser Normalization. Kaiser-Meyer-Olkin Measure of Sampling Adequacy: 0.841. F1 = Factor 1 and F2 = Factor 2

### Internal consistency

The Chronbach’s alpha value was 0.89 (95% CI, 0.88–0.90) from pooled observations of the four rounds: 0.92 in the first (R1), 0.92 in R2, 0.77 in R3, and 0.91 in R4.

### Parallelism

**Fig. 2:**
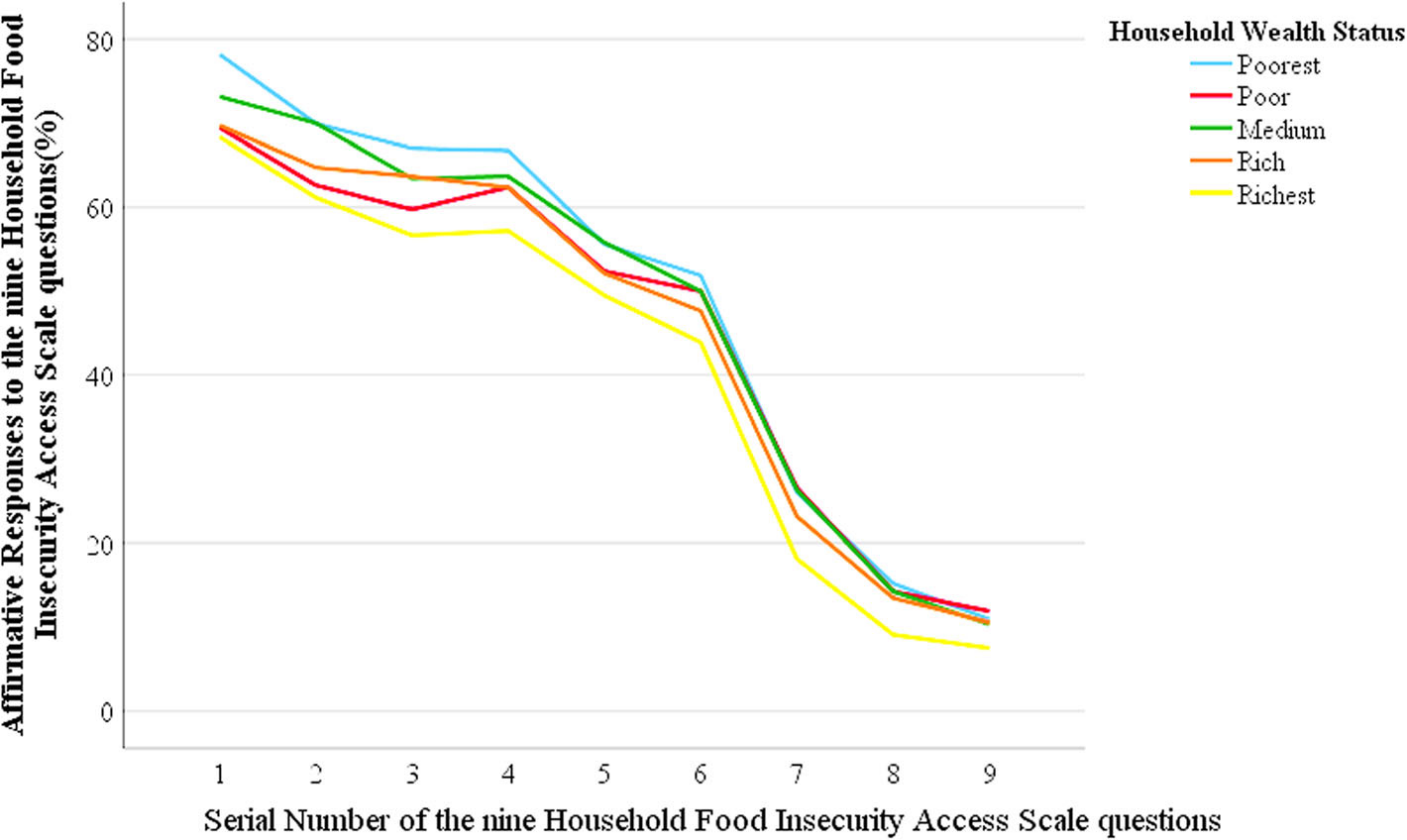
Likelihoods of affirmative responses to the nine HFIAS questions by household wealth strata in rural Wolaita, Ethiopia (pooled observations)

Figure 2 shows the likelihood of affirmative responses across household wealth quintiles. The curves show item responses that were parallel with wealth strata and had an inversely decreasing trend with household wealth.

### Food insecurity severity and household wealth status

This study documented a significant and an inverse dose-response relationship between household wealth and FI severity levels (*P* < 0.01) but the trend was inconsistent between rich and richest strata.

### Food insecurity and food intake

Food intake had a significant and inverse dose-relationship relationship with FI severity levels: pooled observation (*F* = 49.2; *P* < 0.001). In other words, there was a dose-response relationship between food security and the previous day’s consumption of certain food varieties. For example, cereals were consumed in 70.8% of food-secure households but in 62.1% of severely insecure. Similarly higher was the likelihood of consuming vegetables (49.2% vs. 19.6%), fruits (12.1% vs. 3.0%), pulses (23.7% vs. 10.1%), oils (53.1% vs.17.0%), and milk products (18.3% vs. 5.4%). Moreover consumption of cereals, vegetables, fruits, pulses, oils and milk products had an inverse and significant dose-response relationship with FI severity levels (*P* < 0.01).

### Food insecurity over time: reproducibility

The mean HFIAS score at baseline (R1) was 9.01 (95% CI: 8.34–9.67); it was 7.64 (95% CI: 6.97–8.30) in Round 2, 8.29 (95% CI: 7.75–8.84) in Round 3, and 11.77 (95% CI: 11.10–12.44) in Round 4. The pooled mean score for pre-harvest rounds (R1 and R4) was 10.39 (95% CI: 9.92–10.86). It was 7.96 (95% CI: 7.50–8.43) for post-harvest rounds (R2 and R3). As we see consecutive rounds, it significantly decreased in R2 compared with R1 (1.37; 95% CI: 0.39–2.35) and it was higher in R4 than R3. The mean score also varied between pre-harvest and post-harvest seasons (2.42; 95% CI: 1.93–2.92).

## Discussion

This study evaluated the performance of the HFIAS for assessing seasonal variations in FI in the rural Wolaita area in southwest Ethiopia. It mainly assessed internal consistency and construct validity of the tool through repeated observations. The results showed that the tool had satisfactory internal consistency and construct validity across rounds. However, some items required context-sensitive rephrasing, and others required prior discussion about potentially sensitive topics (e.g., food taboos). Moreover, the last three items particularly required skilled interviewers with probing abilities that can curiously pursue to get responses.

The four questionnaire rounds were aligned with agricultural seasons to ensure accurate representation of potential food shortage and access periods. The random selection of lower sampling units and strata of major altitudinal divisions in this study’s population are representative of other Ethiopian settings. Using the same households for repeated surveys, with no loss to follow-up, facilitated accurate estimates of seasonal variations. This study mainly recruited women having young children as respondents to reduce possible response bias.

The overall affirmative responses to the nine HFIAS items were found to be sequentially ordered in increasing severity, except some minor internal deviations. While ‘Worry for food (Q_1_)’ received the highest affirmative responses in all other three rounds, but it was ‘Eat foods that you did not want (Q_4_)’ exceptionally in R3. In the same round, ‘Eat fewer meals in a day’ (Q_6_) had more affirmative responses than its just preceding item. Similarly during round 4, the fourth item (Q_4_) slightly turned up higher than its preceding item. We also documented oscillations in affirmative responses to the items: flipping up in pre-harvest and down in post-harvest rounds (Table 2 and Fig. 1).

Our results showed that affirmative responses decreased with increasing severity of the nine HFIAS questions, which supports Coates et al. [18], but somewhat inconsistent with previous studies in Ethiopia [23], Tanzania [21], and Iran [22] . The first item (‘Worry for food’) had the most affirmative responses in our study in contrast with the second item (‘Unable to eat preferred foods’) previously reported from Butajira district in Ethiopia [23]. The ninth item (‘go day and night without eating’) received the fewest affirmative responses in our study and this was an expected order [18]. However, it was the fourth item (‘Eat foods that you did not want’) that received the least affirmative response in the Butajira study [23]. These deviations could be due to socio-cultural differences. For example, ‘Worry for food’ seemed to reflect an ordinary experience for our respondents, perhaps leading to artificially higher affirmative responses. In contrast to previous Ethiopian studies [20, 23], ‘Eat foods that you did not want’ appeared to have the most affirmative responses in late post-harvest round (R3) in our study. The deviation could be due to differences in interviewing techniques or might be real differences in respondents’ perceptions of food insecurity. Slightly in contrast with previous findings as well as the expected logical sequence, the sixth item (Q_6_) had more affirmative responses than its preceding item (Q_5_) in R3. Similarly in Round 4, the fourth item had slightly more affirmative responses than its preceding item. Accordingly, the first (June) and the fourth (March) rounds could represent the lean season and the middle two rounds to relatively wet season, despite the within variations. During the lean season males often migrate (within or out site the community) for labour works to generate additional income. This season is generally experienced all across the study settings but for longer times (months) in low altitude areas [5, 43]. During this “hunger season” period, household food stocks from the last harvest begin to run out: low production levels, inadequate storage facilities, and accumulated debt all combine to force families to sell or consume their agricultural production well before the new harvest [44].

Our study showed two distinct categorical domains of food insecurity discriminated by factor loadings and total variance explained in response. This was consistent with previous studies in Ethiopia [20, 23]. A similar factor component and equivalent variance in response was reported in rural Tanzania [21] and Lebanon [45] as well as an urban setting in Iran (Tehran) [22]. Yet, inconsistent with Coates et al. suggestion [18] we found no single item alone representing a unique domain. With regards to factor loading, our findings mainly differed from others on the fourth ‘Eat foods that you did not want’, the fifth ‘Eat a smaller meal’, and the sixth ‘Eat fewer meals in a day’ items in the HFIAS. In contrast to our findings, both the fifth and six items indicated severe food insecurity in the rural Tanzania [21] and Lebanon [45] and similarly the sixth item indicated severity domain in Tehran [22]. Unlike ours, the fourth item had no particular domain in a study previously conducted in the Butajira district [23]. These deviations could be due to socio-economic, dietary, and also methodology such as the use of local language version of questionnaire or not, or other contextual differences. This in turn might imply an on-going need to adapt the HFIAS for different settings in Ethiopia.

The HFIAS showed high internal consistency in the study area, particularly in the first, second and fourth survey rounds. A similarly high internal consistency was reported in a previous study in eastern Ethiopia [46]. This was also in line with studies in an urban setting in Iran [22] and rural Tanzania [21]. We however documented a relatively lower Chronbach’s alpha value especially in the third round compared with the other rounds, still this was within the acceptable range [47]. Although these findings are empirically equivalent to a recent finding in Ethiopia [23] and exceed conventional measurements [47], they still raise concerns about internal consistency in tools that use repeated measurements. Therefore, we suggest further longitudinal studies to evaluate and improve internal consistency of the tool. We also recommend further studies assessing comparability of the overall estimates (FI prevalence in this case) from the HFIAS with other indicators.

The current study revealed an inverse dose-response relationship between household wealth and FI. We observed parallelism between FI and household wealth quintile, as reported in previous studies [20–23]. The HFIAS score had an inverse and significant dose-response trend with household wealth status which was as reported in previous studies in Ethiopia and elsewhere [20–23]. Yet, the trend was inconsistent between the wealth strata, possibly due to differences in the state of food security among wealthier households or divergent coping strategies. We constructed wealth index at baseline and assumed less variation across seasons. The higher the FI levels, the lower the dietary intake of the households and vice versa. The result also showed that the consumptions from food groups such as cereals, vegetables, fruits, pulses, oils and milk products was higher among food-secure households compared with severely food-insecure ones. These findings support previous studies in Ethiopia [23, 37].

The proportions of affirmative responses fluctuated across rounds, particularly between pre-harvest and post-harvest seasons. For example, the first six items (Q_1_–Q_6_) received the most affirmative responses in R1 and R4 and the least in the middle two rounds. However, responses to the latter three items (Q_7_–Q_9_) showed slight differences across the first three rounds and tended to peak during the fourth round (Fig. 1). The mean HFIAS scores significantly differed between most consecutive pairs of observations. It also varied between pre-harvest and post-harvest pair of rounds. These findings suggest seasonal variation in household FI. However, the observed difference within the post-harvest rounds was not significant, which might still imply reproducibility of the tool within similar level food insecurity. Similar findings were reported in a recent cross-sectional study in Ethiopia [23].

Some limitations of this study should be considered. The study was conducted in a chronically food-insecure setting, so the affirmative responses could be overestimated if responses were related to intentions to get food aid. We could also expect some under reporting particularly for the last three HFIAS questions pertaining to severe FI conditions due to cultural perceptions that disclosing such extreme situations would be perceived as ‘disobedience to God’. Headey et al. also raise subjectivity related concerns, including likelihood of being prone to response bias on tools such as the HFIAS [19]. Household wealth index was determined at baseline and less variation was assumed across seasons. Thus possible variations in the wealth status across agricultural seasons might have led to different dose-response relationships. We attempted to use the same data collectors across rounds, but it was sometimes difficult to maintain this, which at certain level could have effects on the results. Additionally, we excluded households taking part in government’s PSNP, so it needs evidence as whether these households differ in seasonal FI experiences from the others. This study, for the first time in Ethiopia, tested the performance of the HFIAS for estimating seasonal variations in household FI; we had limited literature for relevant comparison.

## Conclusions

The implications of the seasonality on FI measurements remain underexplored in Ethiopia and thus a valid tool is needed. The HFIAS has been adapted for such use, but few cross-sectional studies have validated it as a measure of FI in Ethiopia. Therefore, the current study evaluated the performance of the tool for measuring FI in rural Ethiopia. We found that the HFIAS could be adapted as a tool for measuring seasonal variations in FI among subsistence households in the study area. However, subjectivities might have inflated affirmative responses to some items indicative of milder FI conditions and vice versa for the severe ones. Moreover, adapting the tool also required meticulous effort to assure contextual validity in terms of cultural food taboos, socio-economic characteristics, and respondents’ access to and expectations about receiving food aid.

## Data Availability

The datasets analyzed during the current study are available from the corresponding author on reasonable request. The data described in this manuscript will also be made publicly available as we complete analysis for the major study.

## Abbreviations

ANOVA: Analysis of variance
CI: Confidence interval
FI: Food insecurity
HDDS: Household Dietary Diversity Scale
HFIAS: Household Food Insecurity Access Scale
